# Role of the left posterior middle temporal gyrus in shape recognition and its reconstruction during drawing: A study combining transcranial magnetic stimulation and functional near infrared spectroscopy

**DOI:** 10.1371/journal.pone.0302375

**Published:** 2024-05-03

**Authors:** Nakako Okamoto, Akitoshi Seiyama, Shota Hori, Satoru Takahashi

**Affiliations:** 1 Department of Nursing, Mental Health & Psychiatric Nursing, Kyoto Tachibana University, Kyoto, Japan; 2 Department of Media Art, Graduate School of Arts-Doctorial Course, Kyoto City University of Arts, Kyoto, Japan; 3 Department of Creative Design & Data Science Center, Akita International University, Yuwa, Akita-City, Akita, Japan; 4 Department of Human Health Science, Graduate School of Medicine, Kyoto University, Kyoto, Japan; 5 Daikin Industries, Ltd., Osaka, Japan; University of Catania Libraries and Documentation Centre: Universita degli Studi di Catania, ITALY

## Abstract

There are numerous reports of enhanced or emerged visual arts abilities in patients with semantic impairment. These reports led to the theory that a loss of function on the language side of the brain can result in changes of ability to draw and/or to paint. Further, the left posterior middle temporal gyrus (l-pMTG) has been revealed to contribute to the higher control semantic mechanisms with objects recognition and integration of visual information, within a widely distributed network of the left hemisphere. Nevertheless, the theory has not been fully studied in neural bases. The aim of this study is to examine role of the l-pMTG on shape recognition and its reconstruction within drawing behavior, by using a combining method of the repetitive transcranial magnetic stimulation (rTMS) and functional near-infrared spectroscopy (fNIRS). Eighteen healthy participants received a low frequency inhibitory rTMS to their l-pMTG during the drawing task of the Benton Visual Retention Test (BVRT). There was a significant decrease of the mean accuracy of reproductions in the Complex designs of the BVRT, compared to the Simple and Medium designs. The fNIRS data showed strong negative correlations with the results of the BVRT. Though our hypothesis had a contradiction that rTMS would have inhibited the brain activity in the stimulated site, the results suggest that shape recognition and its reconstruction such as the BVRT require neural activations of the l-TL as well as that of the l-pMTG.

## Introduction

There are a number of reports of newly developed or enhanced artistic creativity in patients with brain dysfunction (s). In particular, patients suffering from neurological and structural diseases, such as frontotemporal dementia [1], semantic dementia, primary progressive aphasia [2] and Alzheimer’s disease [3], have shown signs of visual arts abilities throughout the progression of their cognitive deterioration. Such cases led to the theory [4, 5] that a loss of function on the language side of the brain results in an enhancement of visual art processing, even though artistic abilities has been suggested to be right hemispheric dominant. However, the theory has not been fully studied in neural bases because of its multifunctional and multiregional processes of drawing.

It has been suggested that the drawing process encompasses visuospatial skills, attention mechanisms, mental representations of space, conceptual knowledge and semantic cognition, and motion planning and control mechanisms [6, 7]. A number of functional neuroimaging studies about drawing behavior have revealed an implication of semantic and phonological networks while drawing, showing a strong bi-hemispheric activation of a widely distributed network of the frontal, parietal, and temporal lobes [8, 9]. A study about drawing by using mental imagery, drawing familiar objects compared to drawing unfamiliar objects (non-objects), showed greater activation in the left inferior temporal cortex, suggesting its involvement in the selection of specific semantic features of the object as well as retrieval of information regarding the perceptual aspects of the object [10]. A clinical report in associative agnosia, the patients showed an inability to copy a very simple figure, even though they were able to retrieve the meaning of the figure [11].There have been other ways of deficits, despite the inability to recognize an object, the patients were able to copy figures accurately, which was usually associated with the brain dysfunction of the left frontotemporal lobe. These reports suggest that, drawing processes involve different neuronal circuits, that processing verbally associable figures and/or non-verbally associable figures. We suppose that, though the language area has not been suggested as the critical region for visual art activities in general, it seems that semantic representations are involved in the processes of drawing, especially in shape recognition and its reconstruction of the verbally associable images (complex figures).

Studies in semantic recognition by using TMS explored an important role of the widely connected network of the left inferior frontal gyrus and the l-pMTG [12]. It has been suggested that there are two distinctive neural structures of semantic processes, for processing weak associations (complex figures) with executive control semantic activation in the l-pMTG, and for processing strong associations (simple figures) with automatic spreading activation in the left angular gyrus [13, 14]. Furthermore, the l-pMTG has been revealed to contribute to higher control semantic mechanisms with manipulation of conceptual knowledge retrieval and the selection of semantic knowledge [15]. Based on these studies, the l-pMTG is possibly involved in shape recognition and its reconstruction during drawing.

The Benton Visual Retention Test (BVRT) has been a reliable tool for both clinical and experimental research, and has been used for many forms of brain impairments and diseases such as Alzheimer’s disease, dementia, aphasia, geriatrics, schizophrenia and children with learning disabilities [16, 17]. The test assesses visual perception, short-term visual memory, and visuo-constructional ability. However, a number of studies that used the BVRT suggested that a verbal mediation component is thought to be associated, due to the use of verbally associable geometric figures ranging from strongly associable images (simple designs. e.g., a pentagon) to weakly associable images (complex designs. e.g., a mixture of geometric figures) [18, 19]. Clinical studies have often reported hearing patients repeat the names of the shapes aloud to themselves as they view the figures before they attempt to produce them, and as they draw them [18]. We adopted this verbal mediation of the BVRT into our experiment, to test shape recognition and its reconstruction during drawing the complex designs of the BVRT.

The aim of this study is to investigate the relationship between the neural activities of specific regions of the l-pMTG and behavioral effects on the shape recognition and its reconstruction during drawing. We employed a combining method of the repetitive transcranial magnetic stimulation (rTMS) and functional near-infrared spectroscopy (fNIRS) [20–22], with the drawing task of the Benton Visual Retention Test (BVRT). rTMS is a noninvasive technique whereby magnetic pulses transiently disrupt neural processing causing behavioral changes [23–25]. By using rTMS, temporally impaired neural situations that supposed to be like patients are created in healthy participants. fNIRS is an optical and wearable neuroimaging techniques that has great advantages of higher time resolutions and make no interference against metallic materials and devices generating electricity or magnetism, which is unlikely to PET and fMRI, and is an ideal candidate for integrated use with TMS, in normal seated positions. We employed rTMS and fNIRS measurements during the drawing test of the BVRT to examine the relationship between neural functions and the behavior.

The aim of the study is to test our hypothesis that inhibitory rTMS to the l-pMTG suppresses the BVRT reproducibility. Furthermore, we discussed the role of the l-pMTG for the shape recognition and its reconstruction during drawing the Complex designs of the BVRT.

## Materials and methods

### Participants

Eighteen healthy volunteers (7 male and 11 female, mean age 27.2 years; range 21–41 years, SD = 5.67, right-handed). Using G*Power 3.1.9.2, pre-analysis for the required minimum sample size resulted in 15 persons, assuming effect size = 0.80, α error probability = 0.05, and power (1-β error) = 0.80. Participants were recruited from undergraduates, graduate students, and people with normal learning ability. All participants were confirmed to have no history of epilepsy, intracranial lesions, medications or alcohol dependence, and no neurological or psychiatric disorders and/or learning disabilities. The recruitment period for the participants in this experiment was from March 1, 2014 to May 31, 2014. Prior to the experiment, all participants provided both written and verbal informed consent. The methods were carried out in accordance with the relevant guidelines and regulations. All experimental protocols were approved by the Kyoto University Graduate School and the faculty of the medical ethics committee (The approval number: Reception No. E1269) and adhered to the tenets of the Declaration of Helsinki.

### BVRT procedure

**Fig 1:**
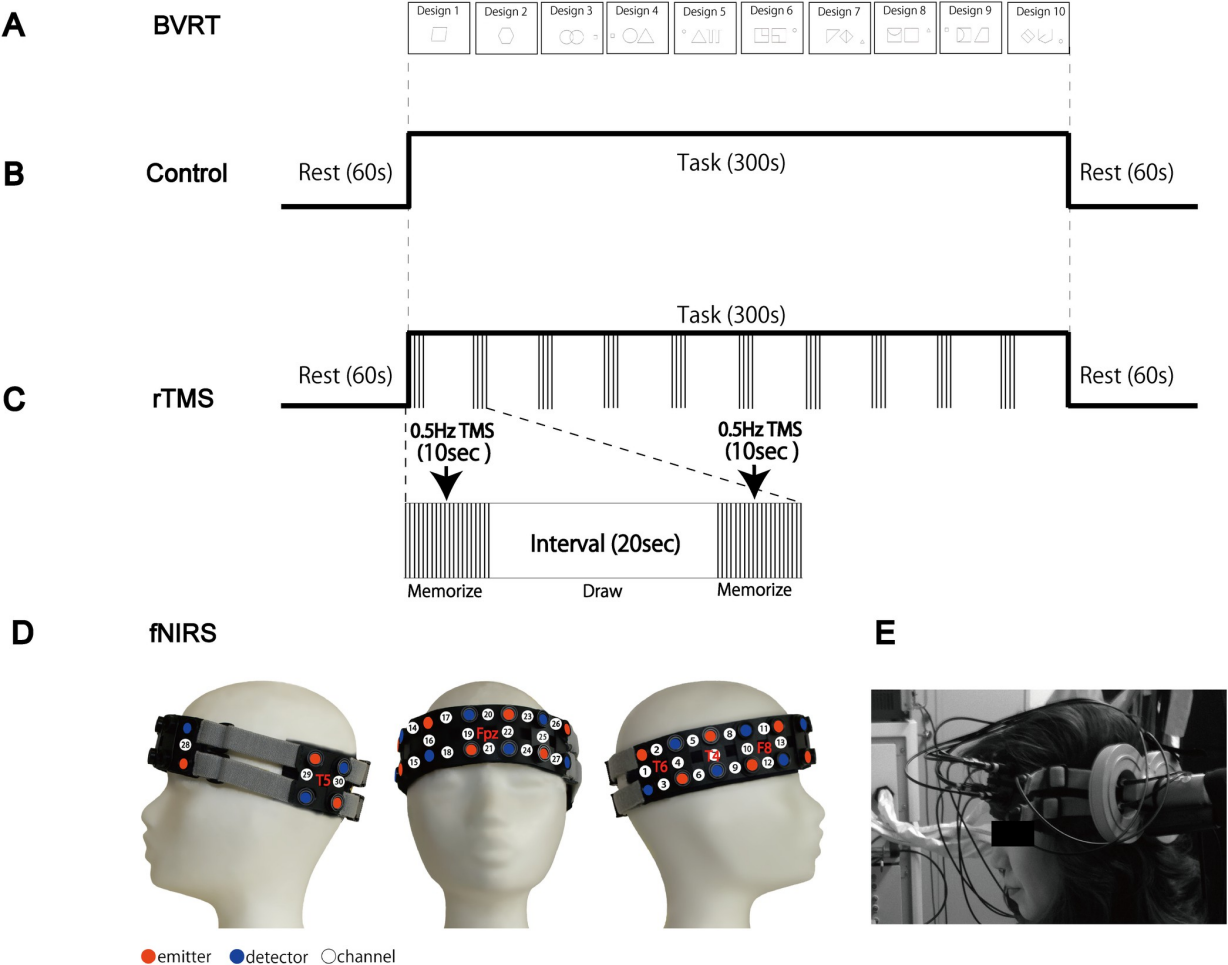
Experimental procedure. **(A)** Ten designs used in the BVRT (One of the two forms). The form consists of ten various designs, starts from simple to complex. The participants were exposed to the design for ten seconds and were then asked to draw them on a sheet of plain paper immediately after the original design was removed. **(B)** Protocol of the control with 0% stimulus output. **(C)** rTMS protocol with a frequency of 0.5 Hz (150 pulses) and a stimulus intensity with 100% of RMT. **(D)** Twelve pairs of emitting and detecting optical fibers were placed based of the International 10–20 system, creating thirty measuring points (channels). **(E)** An 8-figure coil was applied to the l-pMTG, corresponding to the brain area T5 (International 10–20 system).

The tests were conducted individually for each participant in the laboratory. Before the real tests, all participants were exposed to the sample designs in order to understand the procedure. To avoid practice effects, participants were asked to perform two different sets of equivalent forms, which were designated as control and rTMS respectively. The form consisting of ten designs was shown one by one to the participants (Fig 1A). Each design was made up of one or three-figures, consisting of two large central geometric forms and one small peripheral figure, printed on a card (8.5 × 5.5 in.). We employed the method type of Administration A, that is, the participants were exposed to each design for ten seconds and were then asked to reproduce them on a sheet of plain paper immediately after the original design was removed, within twenty seconds in this experiment. Additionally, after the test, participants were interviewed by the examiner about how they remembered the design.

The BVRT scores were assessed by the mean correct scores and the total number error scores with six categories, in accordance with the test manual [26]. The number correct scores are calculated based on an all-or-nothing approach; points are awarded if the reproduction of the design matches the original. The error scores were calculated with six types of major categories such as omissions, distortions, perseverations, rotations, misplacements, and size errors. An analysis on the degree of complexity of the designs was assessed by making a comparison among the Simple designs (the design 1–3), the Medium designs (the design 4–7) and the Complex designs (the design 8–10).

### rTMS protocol

We used a magnetic stimulator (SMN-1200, Nihon Koden, Japan) with an 8-figure coil (YM-131B, Nihon Koden, Japan). A low frequency stimulation of 0.5 Hz rTMS (150 pulses) was applied to the l-pMTG, corresponding to the brain area T5 (International 10–20 system) (Fig 1E). Although many studies have used 1 Hz stimulations as a low frequency rTMS, in this study, we rather employed 0.5 Hz to 1 Hz in order to reduce side effects, such as loud clicking noises from the device. However, 0.5 Hz stimulations has also been found to be effective to inhibit neural activities [27, 28]. Additionally, in control, participants underwent a task with hearing the recorded clicking sound of the rTMS, to reduce the environmental difference of sound affects between control and rTMS.

Prior to the experiment, the stimulus intensity for each participant was determined individually with the minimum intensity of 50 μV with 20% probability in a fully relaxed muscle (resting motor threshold: RMT). We monitored the motor-evoked potential (MEP) of the left first dorsal interosseous muscle by placing the coil over the right primary motor area of the participants. In addition, five of the eighteen participants received the stimulus intensity of 65% of the stimulator output, due to uncomfortable feelings caused by rTMS. Two conditions, control (0% of stimulator output) and rTMS (100% of RMT), were performed on one day with thirty-minute interval. Each task lasted for a total of seven minutes with rest (60 seconds), BVRT (300 seconds), rest (60 seconds) (Fig 1B and 1C).

### fNIRS data acquisition

Brain activity was observed by functional near infrared spectroscopy (fNIRS) (FOIRE-3000, Shimazu Corp, Japan). fNIRS is a non-invasive optical imaging method used to measure changes in the concentration of oxygenated hemoglobin (oxy-Hb) at a depth of 20–30 mm under the scalp [20, 21]. Twelve pairs of emitting and detecting optical fibers were placed on the participant’s head, creating thirty measuring points (channels). The distance between each fiber was 30 mm. The locations of the fNIRS measurement points were determined based on the international 10–20 system for the electrode placements and adjusted with elastic bands to fit each participant’s head (Fig 1D).

A number of base measuring points were set according to the international 10–20 system for each ROIs: the center of the Ch1-Ch4 corresponding to the T6 was set on the right temporal lobe (r**-**TL), the centered point between the Ch20 and the Ch21 corresponding to the Fpz was set to determine the right and left prefrontal cortex (r-PFC; Ch16—Ch19 and l-PFC; Ch22-Ch25), the center of the Ch10-Ch13 corresponding to the F8 was set on the right frontotemporal lobe (r-FTL) and the centered point between Ch29 and Ch30 corresponding to the T5 was set on the left temporal lobe (l**-**TL) [29]. In addition, we observed brain activity on the l-pMTG, where the magnetic coil of the TMS was applied, by passing the fibers through the hole of the coil (Fig 1E).

The total fNIRS measurement time was set at 420 seconds (rest 60—task 300—rest 60) for control and rTMS. The fNIRS data analysis was performed for the 300 seconds (from 0.91 seconds to 299.15 seconds), omitting 60 seconds of the rest before and after the task. The fNIRS data that were outside the software’s acceptable range were automatically deleted. After performing a low-pass filter of 0.01Hz, data were analyzed by using Modified Beer-Lambert Law (S1 Data Raw data of NIRS). In each channel averaged data were obtained without standardized individual data. We calculated an integral value of changes in oxy-Hb for each channel.

### The autonomic nervous system analysis

We assessed the participants’ physical stress by the autonomic nervous system analysis. The autonomic nervous system function was determined by the spectral analysis of heart rate variability, which was measured by pulse oximetry (OLV-3100, Nihon Koden, Japan). All participants were wearing the fiber plug of the pulse oximetry on their left middle finger during the entire experiment. We used the principle of maximum entropy method (MEM) with the program (Map1060, Bio Field, Japan). The heart rate variability’s power spectra were divided into a very low-frequency (VLF) of 0.003–0.040 Hz, a low-frequency (LF) of 0.04–0.15 Hz, and a high frequency (HF) of 0.15–0.40 Hz. In general, HF fluctuations are mediated by both the sympathetic and parasympathetic nervous systems, and LF fluctuations are mediated by the parasympathetic nervous system. The ratio of LF/HF was estimated as the levels of the sympathetic nervous activity representing the participants’ stress conditions. And the ratio of HF/Total (defined as VLF+LF+HF) was estimated as parasympathetic nervous system representing the participants’ relaxed conditions.

### Statistical analysis

Statistical analysis for the BVRT data, a paired t-test was used on the number correct scores and the number error of the BVRT and on the autonomic nervous system function.

For the NIRS data, after confirming normal distribution and homoscedasticity by using the Shapiro-Wilk test and the Kolmogorov-Smirnov test, we conducted a nonparametric analysis of the Mann-Whitney U-Test with the Bonferroni corrections. Data were expressed as mean ±SE. Values of p < 0.05 and p < 0.01 were considered statistically significant. The effect size (Cohen’s d) was calculated.

## Results

### BVRT scores

The BVRT scores were assessed by the mean correct scores and the total number error scores with six categories, in accordance with the test manual [26]. The number correct scores were calculated if the participants accurately drew the designs matched to the original. The error scores were calculated with six types of major categories such as omissions, distortions, perseverations, rotations, misplacements, and size errors. We compared the mean accuracy of the drawings calculated with the correct scores and the total number error scores with six categories of all the participants (*n* = 16/18; two participants were excluded due to an imperfection in the process), between under control and rTMS.

**Fig 2:**
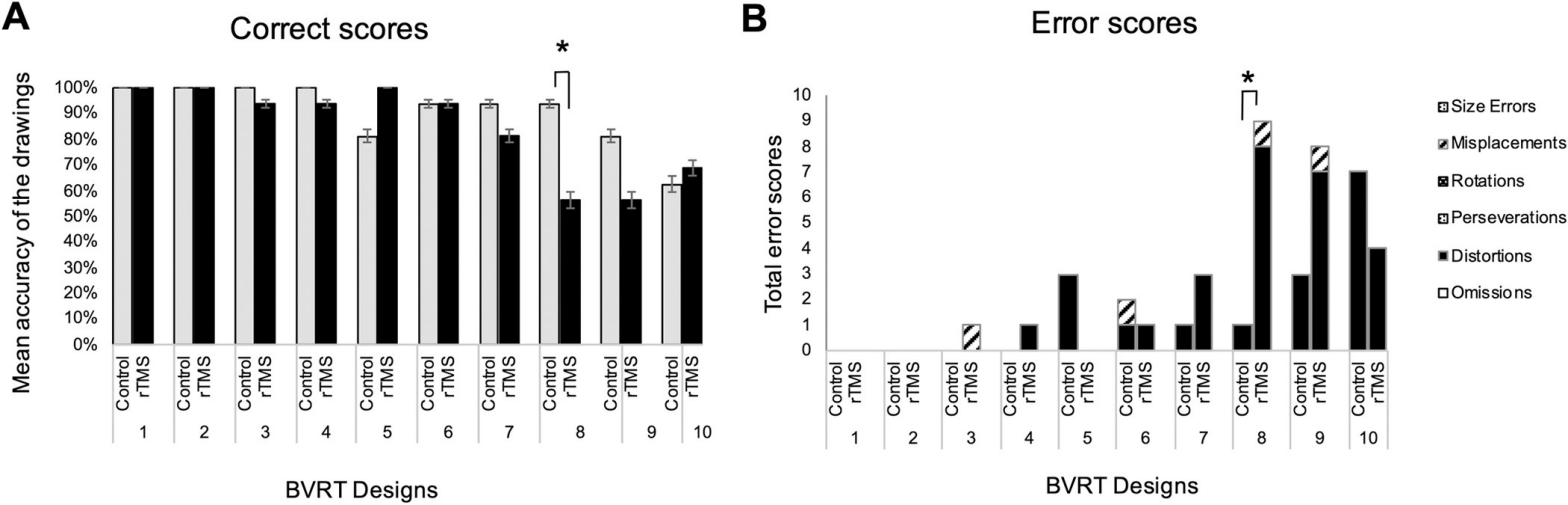
The correct scores and the error scores of the BVRT for control and rTMS. (A)The graph indicates the mean accuracy of the BVRT of all participants under control (gray) and rTMS (black), displayed with percentages. Data are shown as mean +/- SE (*n* = 16). *: *P* < 0.05, (*d* = 0.96). (B)The graph indicates the total error scores of all participants for control and rTMS. Each bar consists of six types of categories such as omissions (white), distortions (black), perseverations (spots), rotations (gray), misplacements (slash) and size errors (check). Data are shown as mean +/- SE (*n* = 16). *: *P* < 0.05, (*d* = -0.95).

In the correct scores, there was a significant decrease in the Design 8 under rTMS (*t* (30) = 2.63, *p* = 0.01, *d* = 0.96) (Fig 2A, S1 Table for BVRT Correct). In the error scores, there was a significant increase of the total error scores in the design 8 under rTMS (*t* (30) = -2.60, *p* = 0.01, *d* = -0.95) (Fig 2B and S2 Table for BVRT Error). Furthermore, there was a significant increase in the “distortions” error scores in the design 8 under rTMS, which often appears in the patients with dementia [30] (*t* (30) = -2.27, *p* = 0.031, *d* = -0.83).

**Fig 3:**
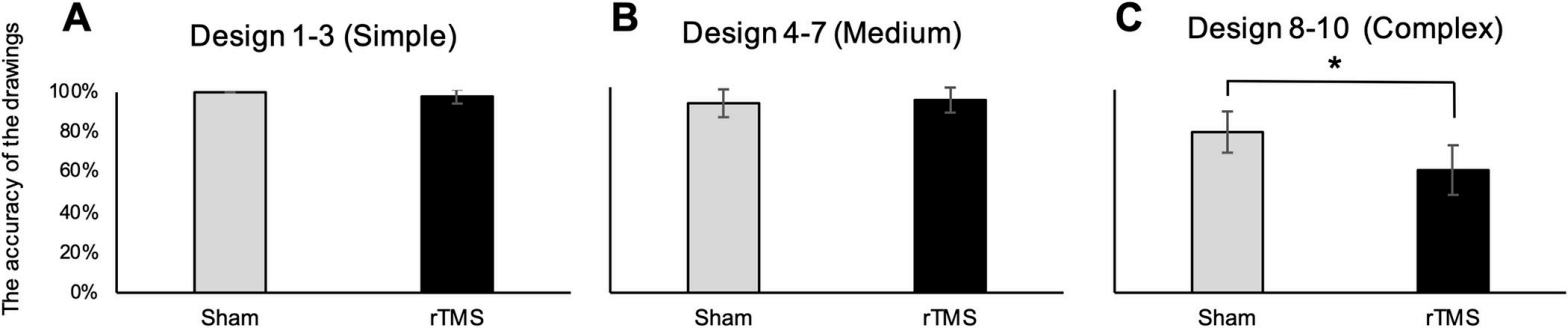
The mean accuracy of the BVRT between under control (gray) and rTMS (black), for the design 1–3 (Simple), the designs 4–7 (Medium) and the designs 8–10 (Complex). Ten designs were divided into three groups, from the design 1 to the design 3 as the Simple designs (A), from the design 4 to the design 7 as the Medium designs (B) and from the design 8 to the design 10 as the Complex designs (C). The graphs indicate the ratio of the mean accuracy of the BVRT of all participants under control (gray) and rTMS (black) displayed with percentages. Data are shown as mean +/- SE (*n* = 16). *: *P* < 0.05, (*d* = 0.42).

Furthermore, we examined the impact of degree of complexity of the designs of the BVRT on participants’ performances of drawings. The BVRT suggested that a verbal mediation component is thought to be associated, due to the use of verbally associable geometric figures ranging from strongly associable images (simple designs) to weakly associable images (complex designs). We compared the mean accuracy of the BVRT drawings of all participants between under control and rTMS, dividing all designs into three groups, the designs 1–3 (Simple), the designs 4–7 (Medium) and the designs 8–10 (Complex). There was a significant decrease in the mean accuracy of the BVRT in the Complex designs under rTMS (*t* (92) = 1.85, *p* = 0.04, *d* = 0.42, Fig 3C), while there were no significant differences in the Simple (Fig 3A) and the Medium designs (Fig 3B).

### Brain activity

**Fig 4:**
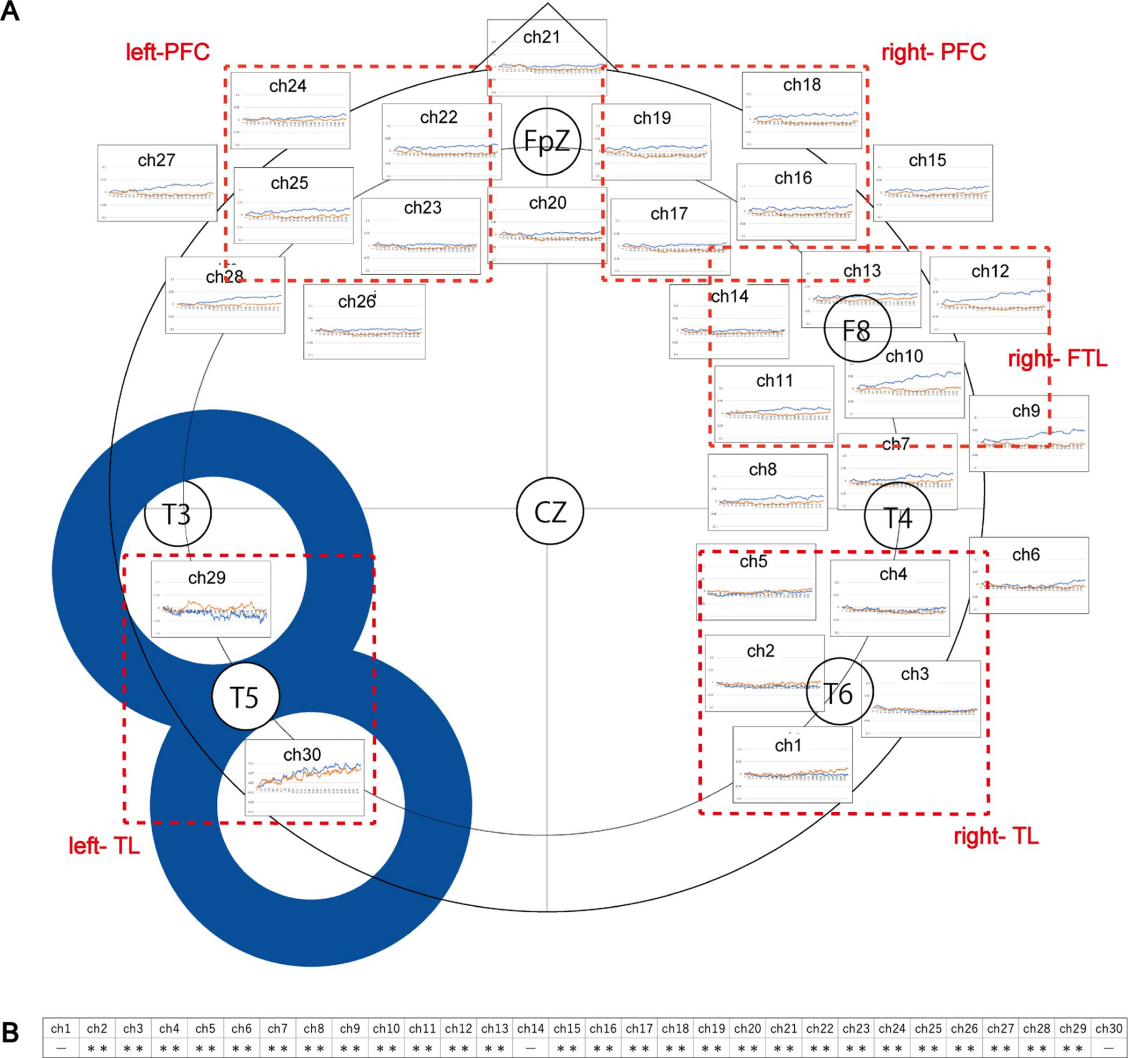
Brain activities under control and rTMS. **(A)**The mean brain activities of the participants for thirty channels between under control (blue) and rTMS (orange). Five regions of interest (ROIs) were selected from the 30 channels, the right temporal lobe (r-TL; Ch1- Ch4), the right frontotemporal lobe (r-FTL; Ch10- Ch13), the right prefrontal cortex (r-PFC; Ch16—Ch19), the left prefrontal cortex (l-PFC; Ch23- Ch25) and the left temporal lobe (l-TL; Ch29 and Ch30). Each region is surrounded by red dotted line. **(B)** Statistical analysis was performed on each channel between control and rTMS. Data are shown as mean (n = 18). *: *P* < 0.05, **: *P* < 0.01 and the "−" denotes no significant.

Brain activity was measured by fNIRS, whereby to assess activations with changes in the concentration of oxygenated hemoglobin (oxy-Hb). Fig 4A shows the mean changes in oxy-Hb for 30 channels. Five regions of interest (ROIs) were defined, the right temporal lobe (r-TL; Ch1- Ch4), the right frontotemporal lobe (r-FTL; Ch10- Ch13), the right prefrontal cortex (r-PFC; Ch16—Ch19), the left prefrontal cortex (l-PFC; Ch23- Ch25) and the left temporal lobe (l-TL; Ch29 and Ch30). We performed a statistical analysis for each channel between under control and rTMS. The results showed a significant decrease in the Ch 2–13 and the Ch15-28 under rTMS. Furthermore, only the Ch29 where was adjacent to the stimulated site, showed a significant increase under rTMS (Fig 4B).

**Fig 5:**
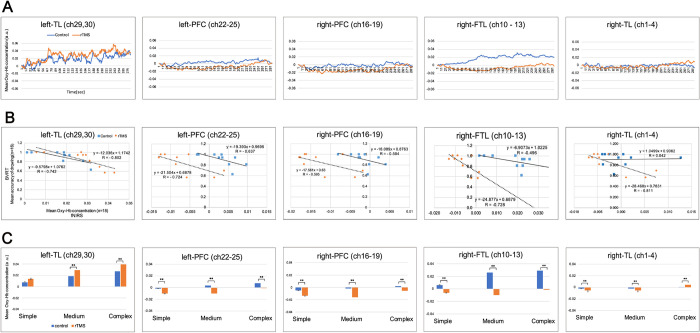
Relationship between BVRT and fNIRS in regions of interest (ROIs). **(A)** Changes of the mean oxy-HB for five ROIs, the right temporal lobe (r-TL; Ch1- Ch4), the right frontotemporal lobe (r-FTL; Ch10- Ch13), the right prefrontal cortex (r-PFC; Ch16—Ch19), the left prefrontal cortex (l-PFC; Ch23- Ch25) and the left temporal lobe (l-TL; Ch29 and Ch30). **(B)** Correlation between two variables of the mean oxy-Hb (n = 18) and their performances of the BVRT (n = 16) for five ROIs. Mean oxy-Hb over a time course of 300 seconds was divided into 10 parts and corresponded to 10 BVRT drawings, and examined the correlation between those two values under control and rTMS. **(C)** An impact by complexity of the BVRT designs on ROIs. The mean oxy-Hb (n = 18) over a time course of 300 seconds during drawings 10 designs was divided into 10 sequences. Data are shown as mean +/- SE (n = 18). **: *p* < 0.01.

Fig 5A shows the mean oxy-Hb under control and rTMS for five ROIs. In the l-TL, the mean oxy-Hb showed a higher percentage than other regions over the time course of 300 sec, both under control and under rTMS (the left graph in Fig 5A). In addition, the mean oxy-Hb was higher in r-FTL under control than in other regions. (the second graph from the right in Fig 5A).

Fig 5B shows correlation between the two variables of the mean oxy-Hb (n = 18) and the mean accuracy of the BVRT (n = 16) under control and rTMS were examined in five ROIs. There were strong negative correlations between two values in the r-TL under rTMS (*r* = - 0.81, *p* < 0.01), the r-FTL under rTMS (*r* = - 0.73, *p* < 0.05), the l-PFC under rTMS (*r* = - 0.72, *p* < 0.05) and the l-TL for both under control (*r =* - 0.74, *p* < 0.05) and rTMS (*r* = - 0.80, *p* < 0.01). Furthermore, there were negative correlation between the two variables in the r-FTL under control (*r* = - 0.50, p < 0.05), r-PFC for both under control (*r* = - 0.50, p < 0.05) and rTMS (*r* = - 0.60, N/S) and the l-PFC under control (*r* = - 0.64, p < 0.05). In addition, there was no correlation between the two variables in the r-TL under control (Fig 5B and S3 Table for correlation).

Furthermore, we examined an impact by complexity of the BVRT designs on ROI. The mean oxy-Hb over a time course of 300 seconds for10 designs was divided into three groups: the Simple (0–90 sec.), the Medium (91–219 sec.) and the Complex (210–300 sec.), and compared between under control and rTMS. As a result, in the right and left PFC and the r-FTL, there were significant decreases of the mean oxy-Hb in all the three groups under rTMS. In the r-TL, there were significant decreases of the mean oxy-Hb in the Simple and the Medium designs, and a significant increase in the Complex designs under rTMS. On the other hand, in the l-TL, there were significant increases of the mean oxy-Hb in the Medium and the Complex designs under rTMS (Fig 5C).

### The autonomic nervous system analysis

From the interviews about the participants’ stress levels from the devices, ten of the eighteen participants stated that they felt certain levels of stress due to restriction by the headsets, and twelve participants reported that they were irritated by the clicking sounds and the flicking feeling of the rTMS. We examined the participants’ stress levels by using the autonomic nervous system analysis. There were no significant differences in the ratio of LF/HF between control and rTMS. Furthermore, there were also no significant differences in the HF/Total between control and rTMS.

## Discussion

The aim of this study was to investigate the implication of the semantic processing of the l-pMTG in drawing behavior, with combining rTMS and fNIRS.

In the result of the BVRT, the total number correct scores of the BVRT showed a significant decrease of the Design 8 under rTMS (Fig 2A), and the total number error scores of the BVRT showed a significant increase of the Design 8 under rTMS (Fig 2B). These results suggest that the rTMS to the l-pMTG have an impact on drawing the Design 8 of the BVRT. The Design 8 is composed of three figures, it is not possible to name them at first glance. We suggest that more linguistic and semantic activities were necessary to recognize and reconstruct the figures. In addition, since the Design 8 was the first design to enter the phase of the Complex designs toward the Design 10, it could have had the greatest impact on participants’ performance.

To examine the relationship between complexities of the BVRT and effects of rTMS, the BVRT results of the 10 designs were analyzed by dividing into three groups, the Simple, the Medium and the Complex design, between under control and rTMS (Fig 3). There was a significant decrease in the mean accuracy of the BVRT in the Complex designs under rTMS (Fig 3C), while there were no significant differences in the Simple (Fig 3A) and the Medium designs (Fig 3B). This suggests that magnetic stimulations to the l-pMTG had an impact on drawing the Complex designs of the BVRT.

The BVRT was developed to assess visual perception, visual short-term memory, and visuo-constructive abilities [26], however, a verbal mediation component is thought to be associated, due to the use of verbally associable geometric figures [18, 19]. In fact, in the present study, seventeen of the eighteen participants reported that they memorized the designs by naming the figures, and they recited those names silently in their mind as they drew them on a sheet of paper. Four participants reported that they remembered the designs as they looked when those designs were very simple, but when the designs became complex, they used verbal association such as “it is a triangle in the square like a face” or “9:00 and 6:45”. In addition, two participants used unique strategies by associating the designs with real life objects, or even creating a short sentence. For example, “A hexagon nut for a bolt” or “The moon is shining on the top-left side of the house”. Furthermore, two participants reported that they felt it more difficult to memorize the Complex designs which included the figures unable to be named. These testimonies of the participants match to the reports of the verbal mediation component of the BVRT.

Furthermore, from the result of specific types of error scores of the BVRT, there was a significant increase in the category of one of the six types of error “distortion” in the Design 8 (the Complex designs), under rTMS (Fig 2B). The reports of patients using the BVRT in Alzheimer’s disease demonstrated the correlation between the severity of impairment and the increase of the number error scores in the types of “omissions” and “distortions”, suggesting impairments of visuospatial cognition function [30, 31]. It is reported that the dementia patients produced disordered composition, less active brush strokes, more facial distortion, and the use of fewer and unnatural colors in their drawings, compared to healthy participants [32]. Another study reported that the drawings made by dementia patients seemed more realistic and precise, or in turn, less realistic and abstract with an exaggeration of particular parts [1]. These reports suggest that semantic representations are involved in the processes of drawing, and impairing these functions might change or enhance visual art activities. We assume that the significant increase of the error type “distortions” under rTMS in this study, could possibly has connections with suppression of semantic processes by rTMS.

In the result of fNIRS, a statistical analysis showed significant differences between control and rTMS at 28 locations. Only the Ch29, which was adjacent to the stimulation site, showed a significant increase under rTMS (Fig 4B). The results contradicted our hypothesis that the 0.5Hz of low frequency rTMS was expected to decrease the oxy-Hb in the stimulated site. These results are not consistent with general efficiency of a low frequency rTMS [33]. Studies combining TMS and NIRS have shown that the oxy-Hb significantly reduced following the low frequency of the 1Hz rTMS, compared to the increases observed in both high frequency of the 2Hz and 5Hz rTMS [34–36]. The possibility of the increased activity of the stimulated site could be related to the cerebral blood flow (CBF). It has been suggested that rTMS affects not only neurons but also the vascular system [20, 37]. We assumed that the BVRT-induced increases of cerebral blood flow (CBF) might have surpassed the rTMS-induced decreases of CBF, which were caused by the effects of hard task conditions and unusual environmental situations. However, the mechanism of rTMS on neurons is still unclear and is expected to be elucidated in the future.

We considered the brain activities between control and rTMS in five ROIs; r-TL, r-FTL, r-PFC, l-PFC and l-TL (Fig 5A). In under control, the mean oxy-Hb showed higher percentage at r-FTL, than at other regions (Fig 5A). It might be possible that performing the BVRT was basically related to the function of the r-FTL, In addition, the mean oxy-Hb in the r-FTL significantly reduced under rTMS. It is curious that rTMS to l-pMTG could have affected the contralateral brain. The r-FTL is located at F8 (10–20 method) and adjacent to the amygdala, which is known for controlling stress and anxiety and/or mood and emotion [38]. In this study, we examined the participants’ stress levels by using the autonomic nervous system analysis. There were no significant differences in the ratio of LF/HF between control a rTMS, suggesting no side effect of stress from rTMS devises.

Furthermore, we tested correlations between the two values of the mean accuracy of the BVRT and the mean oxy-Hb under control and rTMS, for five ROIs (Fig 5B and S3 Table). There was a strong significant negative correlation between those two values in r-TL, r-FTL, l-PFC and the l-TL under rTMS. There were significant negative correlations between the two values in the r-FTL, r-PFC, l-PFC and l-TL under control. The only region did not show the correlation was the r-TL under control. Although the results showed the opposite effects of the rTMS on the stimulated site, the rTMS had stronger impacts on the brain activities and on recognition and its reconstruction of the BVRT. It was suggested that the rTMS to l-pMTG affected not only the stimulated side but also other regions including the contralateral side, resulting in reduced BVRT performance. The broad effects of rTMS in this study might be related to the widely distributed neural network of semantic control functions. Studies about semantic processes suggested that there are two distinctive neural structures for processing weak associations with executive control semantic activation in the l-pMTG, and for processing strong associations with automatic spreading activation in the left angular gyrus [14, 39, 40].

Furthermore, we considered relationship between the mean oxy-Hb and the complexities of the BVRT (the Simple, Medium, and Complex designs), in ROIs. Fig 5C shows that there were significant decreases of the mean oxy-Hb in the three groups under rTMS, in the r-TL, the right and left PFC and the r-FTL, except in the r-TL in the Complex designs under rTMS. In contrast, there were significant increases of the mean oxy-Hb in the Medium and the Complex designs under rTMS, in the l-TL. Furthermore, in l-TL, the accuracy of BVRT decreased as the design became more complex, and the mean oxy-Hb also decreased. These results suggest that there was a connection between the decreases of reproduction and reconstruction of the Complex designs and the rTMS to the l-pMTG. However, it is difficult to explain the direct causal relationship between functions of the l-pMTG and BVRT, because the semantic processing network is assumed to be a broad function that includes not only l-TL but also other areas.

The present study has several limitations. Firstly, the effects of vascular system were not separated from the fNIRS signals. Therefore, it was not entirely clear whether the activation of oxy-Hb under rTMS was due to neural activity or CBF, and further data analysis will be needed. Secondly, there were no other stimulated brain areas that could be compared to the l-pMTG. To clarify the role of l-pMTG in shape recognition and its reconstruction, it would have been better if there were at least two stimulation sites, such as the left angular gyrus. Thirdly, there were no other tasks to assess semantic cognitive functions, such as nonverbal-verbal tasks, naming tasks or word-picture matching tasks. Finally, although this experiment was conducted with an appropriate sample size through power analysis, however, further experiments with larger sample sizes are needed and would be more beneficial to include patients.

Although further investigations are required, the techniques used in this study that combining rTMS and fNIRS with reliable drawing tests are useful to investigate mechanisms of shape and figure recognition during drawing. We believe that this study will lead to clinical research and treatments for patients such as semantic dementia and left temporal lobe dysfunctions. Furthermore, we hope that it will lead to interdisciplinary research in the fields of neurocognitive science, anthropology, and arts.

## Conclusion

We conclude that the inhibitory rTMS to the l-pMTG suppressed the reproducibility of the BVRT, especially the Complex designs.

## Supporting information

S1 Table for BVRT correct The number correct scores are calculated based on an all-or-nothing approach; points are awarded if the reproduction of the design matches the original.
(TIF)

S2 Table for BVRT error The error scores were calculated with six types of major categories such as omissions, distortions, perseverations, rotations, misplacements, and size errors. Integral averaging points.
(TIF)

S3 Table for correlation between fNIRS and the BVRT The table shows the result of correlation between two variables of the mean oxy-Hb (n = 18) and their performances of the BVRT (n-16) under control and rTMS, for five regions of interest (ROIs), the right temporal lobe (r-TL; Ch1- Ch4), the right frontotemporal lobe (r-FTL; Ch10- Ch13), the right prefrontal cortex (r-PFC; Ch16—Ch19), the left prefrontal cortex (l-PFC; Ch23- Ch25) and the left temporal lobe (l-TL; Ch29 and Ch30).
(TIF)

S1 Data S4 NIRS data for control and S4 NIRS data for rTMS. The total fNIRS measurement time was set at 420 seconds (rest 60—task 300—rest 60) for control and rTMS. Smoothing Pinto is 15 points. The fNIRS data that were outside the software’s acceptable range were automatically deleted. After performing a low-pass filter of 0.01Hz, data were analyzed by using Modified Beer-Lambert Law.
(ZIP)
