# Supplementary figures and images for: Role of the left posterior middle temporal gyrus in shape recognition and its reconstruction during drawing: A study combining transcranial magnetic stimulation and functional near infrared spectroscopy
Source: PLoS One. 2024 May 3;19(5):e0302375. doi: 10.1371/journal.pone.0302375 (PMC11068196; doi:10.1371/journal.pone.0302375)

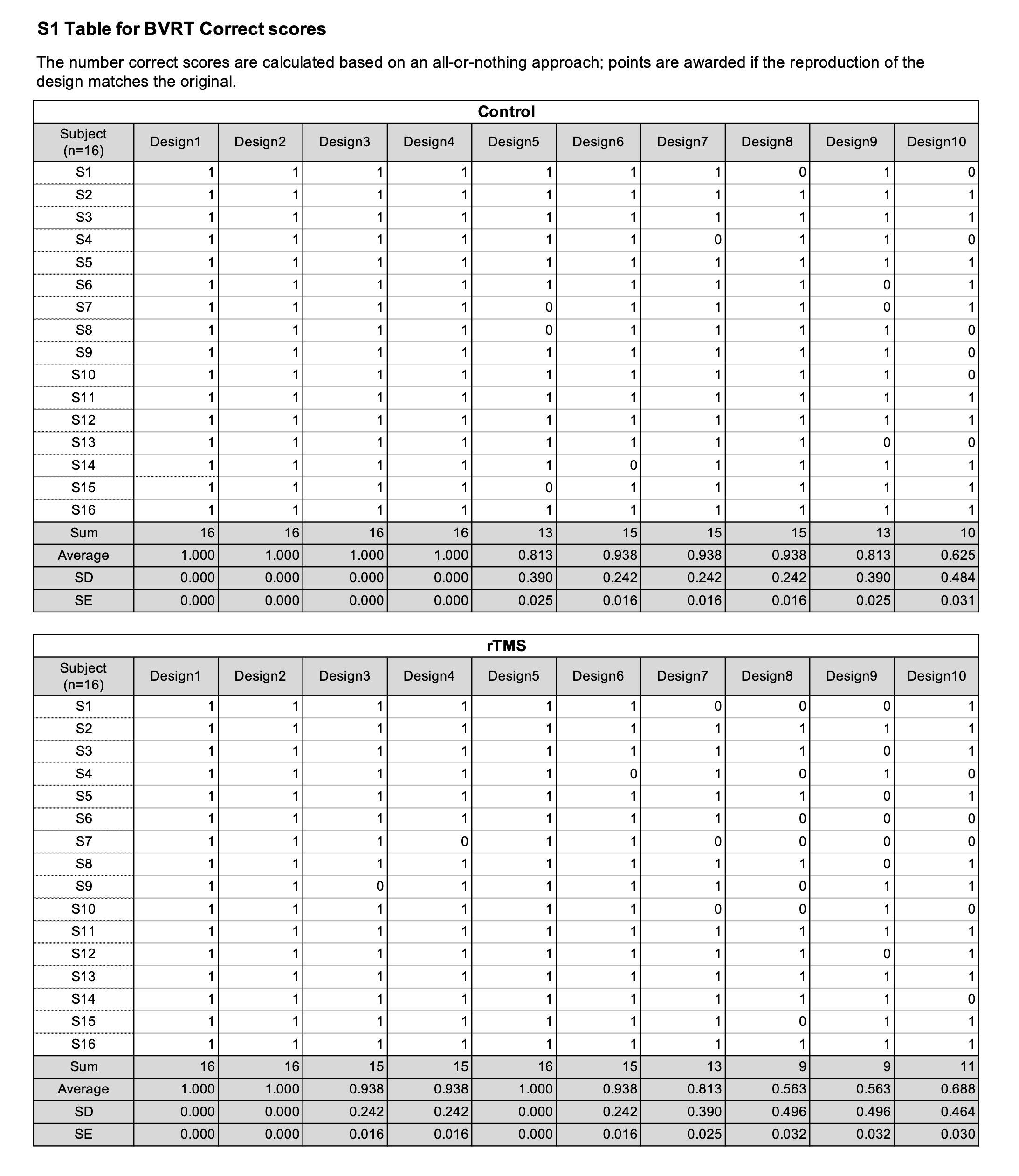

Supplement: S1 Table for BVRT correct — The number correct scores are calculated based on an all-or-nothing approach; points are awarded if the reproduction of the design matches the original. (TIF) [file pone.0302375.s001.tif]

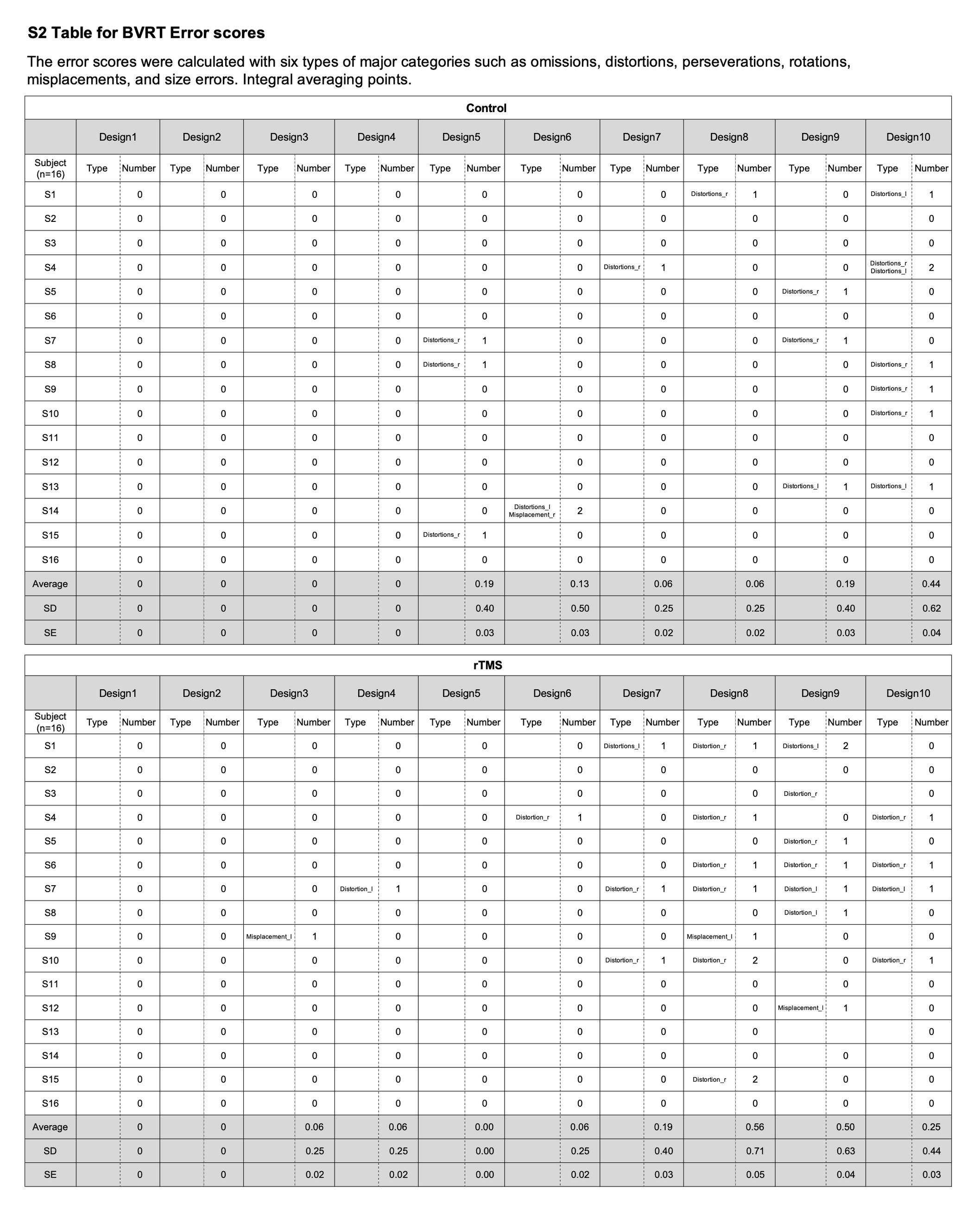

Supplement: S2 Table for BVRT error — The error scores were calculated with six types of major categories such as omissions, distortions, perseverations, rotations, misplacements, and size errors. Integral averaging points. (TIF) [file pone.0302375.s002.tif]

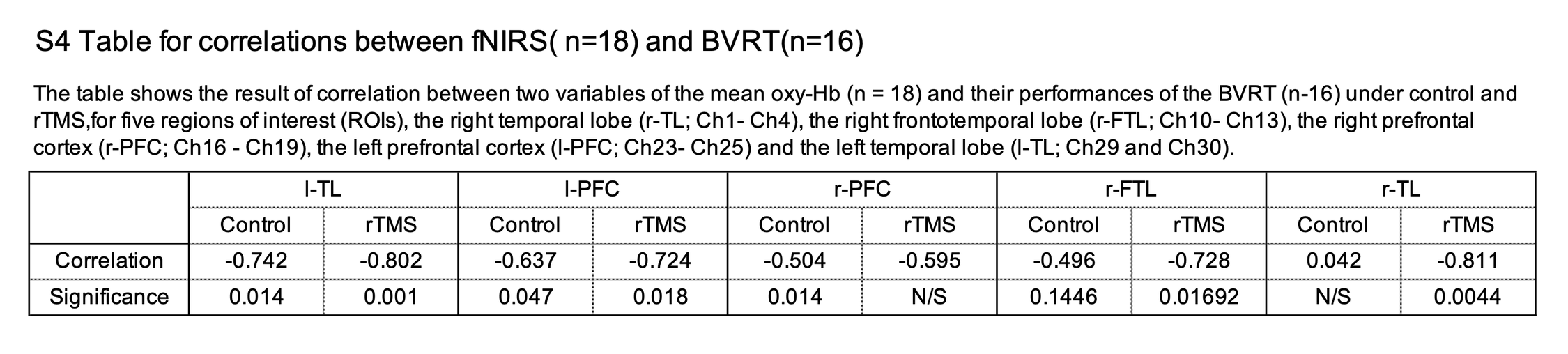

Supplement: S3 Table for correlation between fNIRS and the BVRT — The table shows the result of correlation between two variables of the mean oxy-Hb (n = 18) and their performances of the BVRT (n-16) under control and rTMS, for five regions of interest (ROIs), the right temporal lobe (r-TL; Ch1- Ch4), the right frontotemporal lobe (r-FTL; Ch10- Ch13), the right prefrontal cortex (r-PFC; Ch16—Ch19), the left prefrontal cortex (l-PFC; Ch23- Ch25) and the left temporal lobe (l-TL; Ch29 and Ch30). (TIF) [file pone.0302375.s003.tif]
